# Pyrrolnitrin and Hydrogen Cyanide Production by *Pseudomonas chlororaphis* Strain PA23 Exhibits Nematicidal and Repellent Activity against *Caenorhabditis elegans*

**DOI:** 10.1371/journal.pone.0123184

**Published:** 2015-04-22

**Authors:** Munmun Nandi, Carrie Selin, Ann Karen C. Brassinga, Mark F. Belmonte, W. G. Dilantha Fernando, Peter C. Loewen, Teresa R. de Kievit

**Affiliations:** 1 Department of Microbiology, University of Manitoba, Winnipeg, Manitoba, Canada; 2 Department of Plant Science University of Manitoba, Winnipeg, Manitoba, Canada; 3 Department of Biological Sciences University of Manitoba, Winnipeg, Manitoba, Canada; University of Massachusetts Medical School, UNITED STATES

## Abstract

*Pseudomonas chlororaphis* strain PA23 is a biocontrol agent able to suppress growth of the fungal pathogen *Sclerotinia sclerotiorum*. This bacterium produces an arsenal of exometabolites including pyrrolnitrin (PRN), phenazine (PHZ), hydrogen cyanide (HCN), and degradative enzymes. Production of these compounds is controlled at both the transcriptional and posttranscriptional levels by the Gac-Rsm system, RpoS, PsrA, and the Phz quorum-sensing system. Beyond pathogen-suppression, the success of a biocontrol agent is dependent upon its ability to establish itself in the environment where predation by bacterivorous organisms, including nematodes, may threaten persistence. The focus of this study was to investigate whether PA23 is able to resist grazing by *Caenorhabditis elegans* and to define the role played by exoproducts in the bacterial-nematode interaction. We discovered that both PRN and HCN contribute to fast- and slow-killing of *C*. *elegans*. HCN is well-established as having lethal effects on *C*. *elegans*; however, PRN has not been reported to be nematicidal. Exposure of L4 stage nematodes to purified PRN reduced nematode viability in a dose-dependent fashion and led to reduced hatching of eggs laid by gravid adults. Because bacterial metabolites can act as chemoattractants or repellents, we analyzed whether PA23 exhibited attractant or repulsive properties towards *C*. *elegans*. Both PRN and HCN were found to be potent repellents. Next we investigated whether the presence of *C*. *elegans* would elicit changes in PA23 gene activity. Co-culturing the two organisms increased expression of a number of genes associated with biocontrol, including *phzA*, *hcnA*, *phzR*, *phzI*, *rpoS* and *gacS*. Exoproduct analysis showed that PHZ and autoinducer signals were upregulated, consistent with the gene expression profiles. Collectively, these findings indicate that PA23 is able to sense the presence of *C*. *elegans* and it is able to both repel and kill the nematodes, which should facilitate environmental persistence and ultimately biocontrol.

## Introduction

Successful establishment of a biocontrol agent in a particular environment depends upon a number of factors including competition with indigenous microflora for available resources and resisting the deleterious effects of grazing predators. Bacteria fall prey to a number of organisms; among these, bacterivorous nematodes are thought to play a major role in shaping the microbial community structure. In response, bacteria have developed sophisticated defense strategies to avoid nematode grazing. A small number of bacterial species are capable of forming biofilms on the surface of the nematode, ultimately causing starvation [1,2]. However, a more common mechanism involves the production of exometabolites that have repellent and/or inhibitory effects on nematodes [3,4,5,6,7]. The model organism *Caenorhabditis elegans* is frequently employed for studies of bacterial-nematode interactions. For bacteria that exhibit pathogenicity towards *C*. *elegans*, nutrient availability plays a significant role in determining how these harmful effects are mediated. Under nutrient-limiting conditions, sublethal levels of toxic bacterial metabolites are produced and nematode death proceeds over the course of days. In this case, the so-called “slow-killing” effect is reliant upon bacterial colonization of the nematode intestine [8]. On nutrient-rich media, “fast killing” of the nematodes occurs through intoxication [3,8,9]. For the well-studied pathogen *Pseudomonas aeruginosa*, different mechanisms of fast-killing have been reported depending on the bacterial strain in question and the growth medium employed. Strain PAO1 propagated on brain-heart infusion (BHI) agar causes rapid paralysis and nematode death through HCN poisoning [3]. Whereas on peptone, glucose and sorbitol (PGS) medium, phenazines (PHZ) are responsible for PA14-mediated intoxication [7,8].

Organisms are constantly receiving and responding to chemical signals in their environment and as such, it is not surprising that molecular signalling modulates predator-prey interactions. Studies have shown that bacteria are able to respond to protozoan predator cues by upregulating expression of toxin genes [10,11]. Similarly, bacterial metabolites such as N-acylhomoserine lactone molecules and biosurfactants can act as either chemoattractants or repellents for nematodes [12,13]. This mutual perception and response to chemical signals drives the predator-prey warfare.

*Pseudomonas chlororaphis* strain PA23 is able to protect canola against sclerotinia stem rot caused by the pathogenic fungus *Sclerotinia sclerotiorum* (Lib.) de Bary through a process known as biocontrol [14]. PA23 produces a number of metabolites, many of which are thought to contribute to fungal antagonism including the antibiotics phenazine-1-carboxylic acid (PCA), 2-hydroxy-phenazine (2OH-PHZ) and pyrrolnitrin (PRN), together with hydrogen cyanide (HCN), protease, lipase, and chitinase [15,16,17]. We have determined that PRN is essential for suppression of *S*. *sclerotiorum* [17]. While PHZ play a more minor role in fungal antagonism, they have been found to facilitate PA23 biofilm formation [17]. Production of these metabolites is heavily regulated at both the transcriptional and posttranscriptional levels (S1 Fig). Regulatory elements include the GacS-GacA two component system which functions together with Rsm to positively control antifungal metabolite production [16,18]. Additional regulators include the stationary phase sigma factor RpoS, a sigma regulator called PsrA and the PhzRI quorum-sensing (QS) system [18,19]. Finally, a global stress response known as the stringent response negatively regulates PRN and therefore antifungal activity, primarily through RpoS [20].

At present, the impact of PA23 metabolites on the survival and chemotactic behavior of *C*. *elegans* is unknown. The aim of the current study, therefore, was to determine whether PA23 is able to resist grazing by *C*. *elegans* and to define the role played by PA23 exoproducts in the bacterial-nematode interaction. We discovered that PRN and HCN have repellent and nematicidal activity against *C*. *elegans*. Moreover, co-culturing with *C*. *elegans* leads to altered expression of biocontrol genes and toxic metabolites, suggesting that PA23 is able to detect the presence of this predator and adjust its physiology accordingly.

## Materials and Methods

### Bacterial strains and growth conditions

For a list of bacterial strains and plasmids used in this study see S1 Table. *E*. *coli* was cultured at 37°C on Lysogeny Broth (LB) agar (Difco Laboratories, Detroit, MI). *Pseudomonas* strains were cultured on LB or King’s B (KB) [21] medium at 28°C or in M9 minimal salts medium amended with 0.4% glucose and 1mM magnesium sulfate (MgSO_4_). Antibiotics were used at the following concentrations: ampicillin (Amp; 100 μg/mL), gentamicin (Gm; 15 μg/mL), tetracycline (Tc; 15 μg/mL) for *E*. *coli*, and piperacillin (Pip; 40 μg/mL), Gm (20 μg/mL), Tc (15 μg/mL) for PA23. All antibiotics were obtained from Research Products International Corp. (Mt. Prospect, IL).

### Nematode strain and culture conditions

The *C*. *elegans* strain used in this work was wild-type Bristol N2 which was maintained at 15°C on nematode growth medium (NGM) [22] inoculated with *E*. *coli* OP50. Synchronous cultures were produced according to the protocols available in NematodeBook [23]. L4-stage hermaphrodites were used in the studies described herein.

### Nucleic acid manipulation

Standard techniques were employed for purification, cloning and other manipulations of DNA [24]. Polymerase chain reaction (PCR) was performed following standard conditions suggested by Invitrogen Life Technologies data sheets supplied with their *Taq* polymerase.

### Creation of a PA23 *hcn* mutant

The PA23 *hcn* mutant strain was generated as follows. A portion of the PA23 *hcn* gene cluster was PCR amplified using primers hcnA-FOR and hcnC-REV (Table 1). Primers were designed from the *Pseudomonas fluorescens* CHA0 *hcnABC* gene sequence (accession no. AF053760). A TOPO kit (Invitrogen Life Technologies, Burlington, Ont.) was used to clone the 1.9-kb PCR product into the pCR2.1-TOPO vector generating pCR*hcnABC* ^*’*^-23. pCR*hcnABC* ^*’*^-23 was digested with *Hin*dIII and *Xho*I, and the 1.9-kb insert was subcloned into the same sites of the suicide vector pKNOCK-Tc [25]. Triparental mating between the donor [*E*. *coli* DH5α λpir (pKNOCK*hcnABC* ^*’*^-Tc)], helper [*E*. *coli* DH5α(pRK600)] and recipient (PA23) was performed to insertionally interrupt the wild-type *hcnABC* gene cluster. The *hcn* mutation in PA23*hcn* was confirmed by PCR and testing for a lack of HCN production using Cyantesmo paper (Machery-Nagel GmbH & Co., Germany).

**Table 1:** Behaviour and pathological symptoms of *Caenorhabditis elegans* (N2) on lawns of PA23 and derivative strains at 48h on nematode growth medium.

Strains	Growth defects^1^ (%)	Number of eggs laid^2^	Egg hatch (%)^2^	Colonization^3^	Swollen tail (%)^3^	Enlarged excretory canals (%)^3^	Disintegrated gonads (%)^3^
PA23	45.2 (5.0)	260 (20)	100	extensive^a^	28.3 (1.5)	39.7 (2.5)	22.8 (1.6)
Δ*phz*	63.2 (10)	210 (30)	69.1 (7.0)	extensive^a^	47.7 (2.5)	39.8 (2.3)	29.8 (1.3)
Δ*prn*	25.4 (5.0)	275 (25)	100	mild^b^	0	0	20.7 (2.7)
Δ*prn*/*phz*	26.5 (5.0)	278 (20)	100	mild^b^	0	0	17.7 (3.3)
Δ*hcn*	22.3 (2.5)	328 (30)	100	mild^b^	12.6 (2.7)	21.3 (2.9)	0
AI-deficient	none	953 (50)	100	none	0	0	0
Δ*phzR*	none	1003 (45)	100	none	0	0	0
Δ*rpoS*	58.0 (4.5)	221 (20)	77.4 (4.5)	extensive^a^	41.1 (4.0)	30.4 (3.8)	26.8 (3.3)
Δ*psrA*	55.1 (5.0)	225 (25)	84.4 (4.4)	extensive^a^	44.7 (4.6)	29.8 (3.3)	21.6 (4.5)
Δ*gacS*	none	1160 (92)	100	none	0	0	0
*E*. *coli* OP50	none	1307 (101)	100	none	0	0	0

^1^Growth defects include growth arrest of the original L4-stage subjects or the L1/L2-stage progeny; mean (SD) obtained from a triplicate set.

^2^Mean (SD) obtained from three replicates examining 5 adult hermaphrodites for each.

^3^Thirty nematodes were examined in each trial; mean (SD) obtained from three trials.

^a^extensive—colonization throughout the entire gut.

^b^mild—colonization in only specific areas of gut, either upper or lower intestine.

### *Caenorhabditis elegans* slow- and fast-killing assays

*Caenorhabditis elegans* slow-killing assays were performed by spotting 10 μl of a 1/10 dilution of an overnight bacterial culture grown in NGM broth onto a 35x10 mm NGM agar plate. After 24 h incubation at 28°C, the plates were cooled to room temperature and seeded with 25 to 30 L4-stage nematodes. The plates were then incubated at 25°C and the nematodes were scored for viability by examining nematodes with a stereomicroscope over a ten-day period. Nematodes were considered dead if they did not respond to touch with a nematode pick or tapping of the assay plate against the stereomicroscope stage. Three replicates were included for each trial and the assays were repeated three times. Fast-killing assays were executed in a similar manner except that BHI agar was used instead of NGM agar and the nematodes were monitored every hour for 9 h.

### Effect of purified PRN on *Caenorhabditis elegans* viability and egg hatching

All assays were conducted in 96-well culture plates. *C*. *elegans* L4-stage nematodes and eggs were collected separately in sterile water; approximately 20 nematodes and 15 eggs were used per well. Nematodes and eggs were incubated at 25°C in purified PRN (Sigma, St. Louis, MO) at the following concentrations: 0 μg/ml (water control), 0.1, 0.5, 1.0, 5.0 and 10 μg/ml. Nematode viability was assessed at 1, 3, 6, 12, 18, 24, 48 and 72 h and percent egg hatch was determined at 1, 3, 6, 24 and 48 h. Five replicate wells were used per trial and the assays were repeated three times. One representative data set is shown.

### Chemotaxis Assays

Overnight cultures grown in NGM broth were diluted 10-fold and 10-μl volumes of the two bacterial strains being tested were spotted equidistant from a central point on a 60x15 mm NGM agar plate. Plates were incubated for 24 h at 28°C to allow for bacterial growth. To obtain synchronous L4-stage nematodes, 5 adult hermaphrodites were transferred to NGM agar plates spotted with *E*. *coli* OP50 and allowed to lay eggs. After flame-killing the adults, plates were incubated at 15°C for 4 days to allow the nematodes to reach the L4-stage. Nematodes were collected in M9 buffer, and a 20-μl aliquot (containing 50–100 nematodes) was spotted onto the centre of each plate. The number of nematodes on the two bacterial colonies was counted 24 h after transfer. The chemotaxis index was calculated based on the formula = {(number on spot 2—number on spot 1)/total number of nematodes on spot 1 + spot 2}.

### Generation of bacteria expressing the mCherry red fluorescent protein

Because plasmid pMCh-23 contains the mCherry red fluorescent protein gene, bacteria harboring this plasmid are easily visualized under the fluorescence microscope [26]. pMCh-23 was electroporated into PA23, the Δ*prn*, Δ*phz*, Δ*prn/phz*, Δ*hcn*, Δ*phzR*, Δ*rpoS* and Δ*psrA* mutants as well as the AI-deficient PA23(pME6863). mCherry RFP was visualized using 587 nm excitation and 610 nm emission wavelengths.

### Microscopic imaging of *Caenorhabditis elegans*

NGM plates were spotted with the aforementioned bacterial strains harboring pMCh-23 and incubated overnight at 28°C. Plates were cooled to room temperature prior to seeding with nematodes, followed by incubation at 25°C. For microscopic examination, nematodes were mounted on 2% agarose pads on glass microscope slides and anesthetized with 10 mmol/L Levamisole (Sigma) in M9 buffer. Nematodes were examined with a Zeiss LSM 700 scanning confocal laser microscope and a Zeiss Observer Z1 inverted microscope (Carl Zeiss Microscopy GmbH, Göttingen, Germany).

### Generation of *gacA-lacZ*, *gacS-lacZ*, and *psrA-lacZ* transcriptional fusions

To construct a *gacA-lacZ* transcriptional fusion, the promoter region of *gacA* was PCR amplified using primers nGacAtrans-FRW and nGacAtrans-REV. The 565-bp product was cloned into pCR2.1 (pCR*gacA*up). pCR*gacA*up was digested with *Hin*dIII and *Sma*I and the insert was subcloned into the same sites of pLP170, creating pGACA-*lacZ*. To generate the *gacS-lacZ* fusion, the *gacS* promoter region was PCR amplified using primers newGacStrans-FRW and newGacSR-trans-REV. The 480-bp product was cloned into pCR2.1 (pCR*gacS*up), and then excised with *Sma*I and *Bam*HI and cloned into the same sites of pLP170, generating pGACS-*lacZ*. To construct a *psrA-lacZ* transcriptional fusion, the primers psrAFOR and psrAREV were used to amplify a 948-bp product which was cloned into pCR2.1 (pCR-*psrA*). The promoter region of *psrA* was amplified using primers M13-REV and psrA*Bam*HI-REV and pCR-*psrA* as the template DNA. The 870-bp product was digested with *Bam*HI and the insert was cloned into *Sma*I/*Bam*HI-digested pLP170, generating pPSRA-*lacZ*.

### Analysis of transcriptional fusions in the presence and absence of *Caenorhabditis elegans*

The activity of *prnA-*, *phzA-*, *phzI-*, *phzR-*, *gacA-*, *gacS-*, *rpoS- and psrA-lacZ* transcriptional fusions was determined in PA23 cultured in the presence and absence of *C*. *elegans*. Nematodes were collected in M9 buffer and ~200 were added to PA23 carrying each of the *lacZ* fusion plasmids. Cells were grown at room temperature (22–23°C) for 24, 48 and 72 h in M9 minimal medium supplemented with 1 mM MgSO_4_ and 0.2% glucose prior to analysis of *β*-galactosidase activity [27].

### Hydrogen cyanide gene expression analysis

To determine whether *hcnA* is quorum sensing controlled, expression of an *hcnA-lacZ* translational fusion on pME3219 was measured in PA23 and PA23*phzR*. Both strains were grown in M9 minimal medium (1mM MgSO_4_ and 0.2% glucose) for 24 h prior to analysis of *β*-galactosidase activity.

### Antifungal assays

To assess the ability of PA23 grown in the presence and absence of the nematodes to inhibit the growth of *S*. *sclerotiorum in vitro*, a radial diffusion assay was performed as described by Poritsanos *et al*. [16]. Five replicates were analysed for each strain and the experiments were repeated three times.

### Protease analysis

Extracellular protease activity was determined by inoculating 5 μL of a 72 h culture onto 2% skim milk agar plates. Zones of lysis were observed around the colony after 24–36 h growth at 28°C [16]. Data represent the average of five replicates and the assay was repeated three times.

### Acyl homoserine lactone signal analysis

Total autoinducer production was monitored according to Ling *et al*. [28], with the following modifications. PA23 was grown in the presence or absence of *C*. *elegans* for 72 h at room temperature in 30 ml M9 minimal media supplemented with 1 mM MgSO_4_ and 0.2% glucose. Cells were pelleted and cell-free supernatants were extracted twice with an equal volume (30 ml) of acidified ethyl acetate. The ethyl acetate fractions were pooled and concentrated to a final volume of 1 ml. For AHL quantification, 100 μl aliquots of each extract were tested according to Selin *et al*. [19]. Samples were analysed in triplicate and the experiments were repeated twice.

### Motility analysis

Flagellar (swimming) motility was monitored according to Poritsanos *et al*. [16]. For the assays, five replicates were analysed and the experiment was repeated three times.

### Quantitative analysis of phenazine and pyrrolnitrin production

PA23 cultures were grown in the presence and absence of the nematodes at room temperature in 30 ml M9 minimal medium supplemented with 1 mM MgSO_4_ and 0.2% glucose. Cultures were allowed to grow for 72h before being subjected to PHZ extraction [17]; PRN extractions were performed after 5 days of growth. Quantification of PRN by HPLC followed the protocol of Selin *et al*. [17], with the following modifications. Toluene was added to the culture supernatants as an internal control. Peaks corresponding to the toluene and PRN were analysed by UV absorption at 225 nm using a Varian 335 diode array detector. For both the PHZ and PRN analysis, samples were analysed in triplicate and the experiments were repeated twice.

### Statistical analysis

An unpaired Student’s *t* test was used for statistical analysis of PHZ, PRN, and AHL production, swimming motility, antifungal activity and protease production. The Bonferroni test was applied to determine the chemotactic preference of nematodes for PA23 and derivative strains. The log-rank (Mantel-Cox) test was applied for statistical analysis of pairwise comparisons in the fast and slow killing assays.

## Results

### Cyanide is the primary metabolite responsible for rapid killing of *Caenorhaditis elegans* by PA23

**Fig 1:**
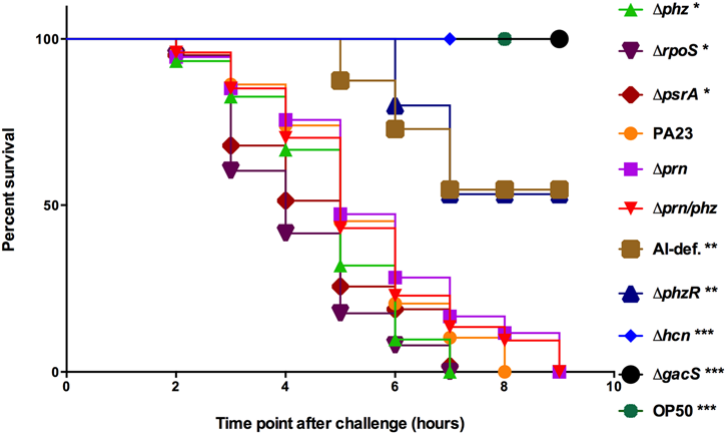
Fast-killing of *Caenorhabditis elegans* by *Pseudomonas chlororaphis* PA23. Kaplan-Meier survival plots of *C*. *elegans* N2 (*n* = 25) fed *E*. *coli* OP50, PA23 or derivative strains propagated on BHI agar. Nematode viability was monitored every hour for 9 hours. Each data point represents the average of three biological replicates. Experiments were performed three times; one representative data set is shown. Asterisks indicate significant difference from the wild type as determined by the log-rank test (*, P<0.05; **, P<0.01; ***, P<0.001).

Pseudomonads can cause *C*. *elegans* lethality via two non-mutually exclusive mechanisms known as fast and slow killing. We were interested to learn whether PA23 exhibited lethality to *C*. *elegans* through one or both of these means. In fast-kill assays, no toxicity was observed with either the *hcn* or *gacS* mutant (Fig 1). The *hcn* mutant does not produce HCN, while the *gacS* mutant is completely devoid of toxic metabolites including PRN, PHZ and HCN [16]. The QS-deficient strains, PA23*phzR* and PA23 (pME6863), exhibited intermediate nematicidal activity with only 50% of the nematodes viable at 9 hours. We have previously reported that these strains produce markedly reduced PHZ and PRN [19]. When these strains were analyzed for HCN production in the current study, only a low amount of this compound was produced (S2 Fig). These findings are supported by *hcnA-lacZ* analysis where expression levels were five-fold lower in the *phzR* mutant (856.4 ± 57.6) compared to the wild type (4967.4 ± 437.9). The residual HCN expression likely accounts for the increased nematicidal activity associated with the QS-deficient strains compared to the *hcn* mutant. There was no difference in the rate of killing of the *prn* knock out mutants PA23-8 (Δ*prn*) and PA23-63-1 (Δ*prn/phz*) compared to the wild type (Fig 1). Interestingly, the highest rate of mortality was observed when nematodes were fed the *phz*, *rpoS* and *psrA* mutants, with 100% mortality occurring at 7 hours (Fig 1). All three strains have been found to produce elevated PRN [17,18,20]. Collectively these findings indicate that HCN is the primary metabolite responsible for fast killing of *C*. *elegans* by PA23 grown on BHI media; however, at levels over and above that of wild type, PRN increases the rate of killing.

### Slow killing assays

**Fig 2:**
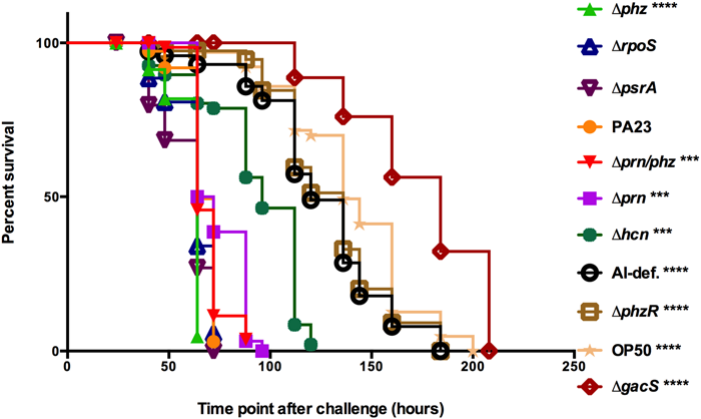
Slow-killing of *Caenorhabditis elegans* by *Pseudomonas chlororaphis* PA23. Kaplan-Meier survival plots of *C*. *elegans* N2 (*n* = 25) fed *E*. *coli* OP50, PA23 or derivative strains propagated on NGM agar. Nematodes were assessed for viability over a ten-day period. Each data point represents the average of three biological replicates. Experiments were performed three times; one representative data set is shown. Asterisks indicate significant difference from the wild type as determined by the log-rank test (***, P<0.001; ****, P<0.0001).

In contrast to fast killing, slow killing is brought about by culturing bacteria on low-nutrient media which doesn’t support production of high levels of toxic compounds. Under these conditions, death or disease is mediated by bacterial colonization of the *C*. *elegans* gut. As illustrated in Fig 2, when we assayed PA23 and derivative strains for their slow-killing effects, the highest degree of lethality was observed when nematodes were grown on PA23-63, which produces 2.2 times as much PRN as the wild type [17]. For the *rpoS* and *psrA* mutants which produce approximately 1.5 times as much PRN as PA23 [18,20], there was no significant difference in killing. The *prn* mutants PA23-63-1 (Δ*prn/phz*) and PA23-1 (Δ*prn*), and the *hcn* mutant showed reduced lethality, resulting in 100% mortality at 88, 96 and 120 hours, respectively (Fig 2). Viable nematodes were observed at 160 hours when growing on the QS-deficient strains and even longer (184 h) on the *gacS* mutant (Fig 2).

To better understand how PA23 affects nematode physiology, microscopic analysis of *C*. *elegans* propagated on bacteria labelled with the fluorescent reporter mCherry was undertaken. Initially, L4-stage nematodes were assessed for growth and fertility defects under low magnification. When cultured on the *phz* mutant, growth was dramatically inhibited with 63.2% of the nematodes arrested at the L4 stage compared to 45.2% for the wild type (Table 1). More modest growth delays were observed when propagated on the Δ*prn* (25.5%), Δ*prn/phz* (26.5%) and Δ*hcn* (22.3%) strains (Table 1). On the *rpoS* and *psrA* mutants, which produce elevated PRN, growth inhibition was 58% and 55%, respectively. No growth aberrations were noted when either the QS-deficient strains or the *gacS* mutant were provided as the food source (Table 1). *C*. *elegans* fertility defects were monitored by assessing the quantity of eggs produced as well as the hatching frequency. We discovered that growth on bacteria producing PRN, PHZ, or HCN, characterized by the wild type and the *phz*, *prn*, *prn/phz*, *hcn*, *rpoS* and *psrA* mutants, lead to a 4- to 5-fold reduction in the number of eggs laid compared to growth on *E*. *coli* OP50 (Table 1). On the *gacS* mutant and QS-deficient strains, which do not produce these compounds, high numbers of eggs were produced. Collectively, these findings indicate that PA23 exoproducts reduce *C*. *elegans* egg production. With respect to egg hatch frequency, only bacteria overproducing PRN (Δ*phz*, Δ*rpoS* and Δ*psrA*) showed less than 100% hatch (Table 1).

### The effect of purified pyrrolnitrin on *Caenorhaditis elegans* viability and egg hatch frequency

**Fig 3:**
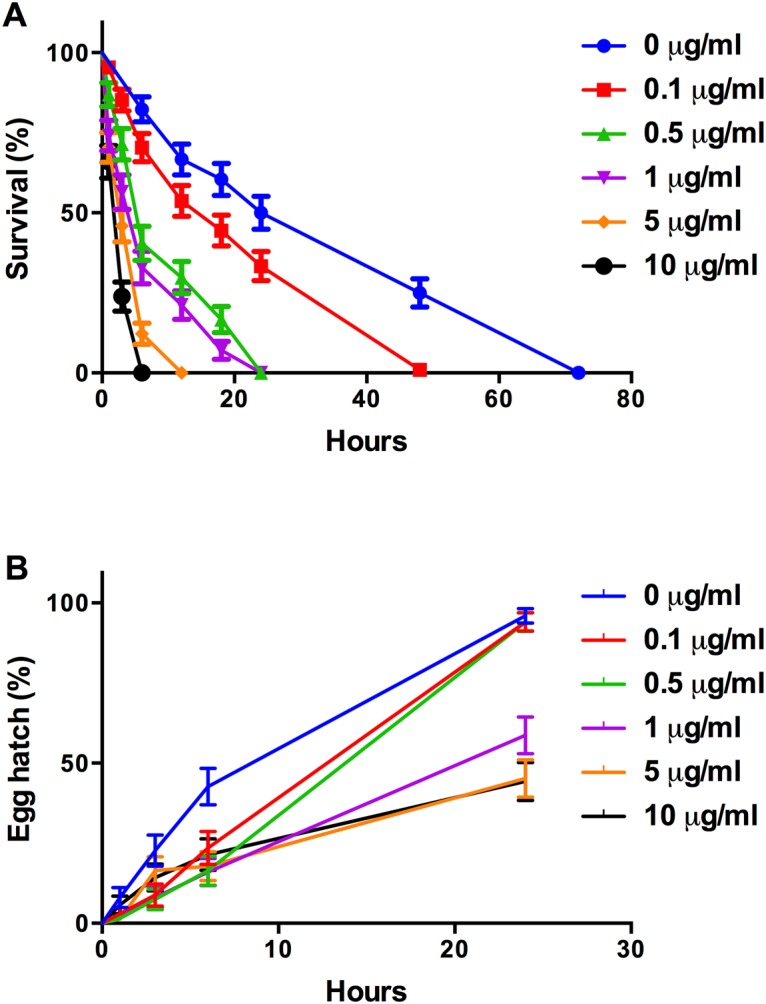
Pyrrolnitrin reduces *Caenorhabditis elegans* viability and egg hatching. *C*. *elegans* L4-stage nematodes and eggs were incubated in microtitre dishes containing purified PRN at the following concentrations: 0 μg/ml (water control), 0.1, 0.5, 1.0, 5.0 and 10 μg/ml. (A) Nematode viability was assessed at 1, 3, 6, 12, 18, 24, 48 and 72 h; (B) percent egg hatch was determined at 1, 3, 6, 24 and 48 h. Five replicate wells were used per trial and the assays were repeated three times. One representative data set is shown.

To further establish a role for PRN in *C*. *elegans* lethality, we exposed L4-stage nematodes to purified PRN at concentrations ranging from 0.1 to 10 μg/ml consistent with PRN levels produced by PA23. As outlined in Fig 3A, in the presence of 0.1 μg/ml PRN, all of the nematodes were dead by 48 hours versus 72 hours for the control. The percent survival continued to decrease in a dose-dependent fashion underscoring the nematicidal effects of PRN on *C*. *elegans*. In terms of egg hatching, exposure to lower concentrations of PRN (0.1 and 0.5 μg/ml) extended the time required for the eggs to hatch. Whereas exposure to higher PRN concentrations (1.0–10.0 μg/ml) reduced hatching to less than 50% after 24 h exposure (Fig 3B); this remained unchanged at 48 h (data not shown).

### Microscopic analysis of *Caenorhaditis elegans* feeding on PA23

**Fig 4:**
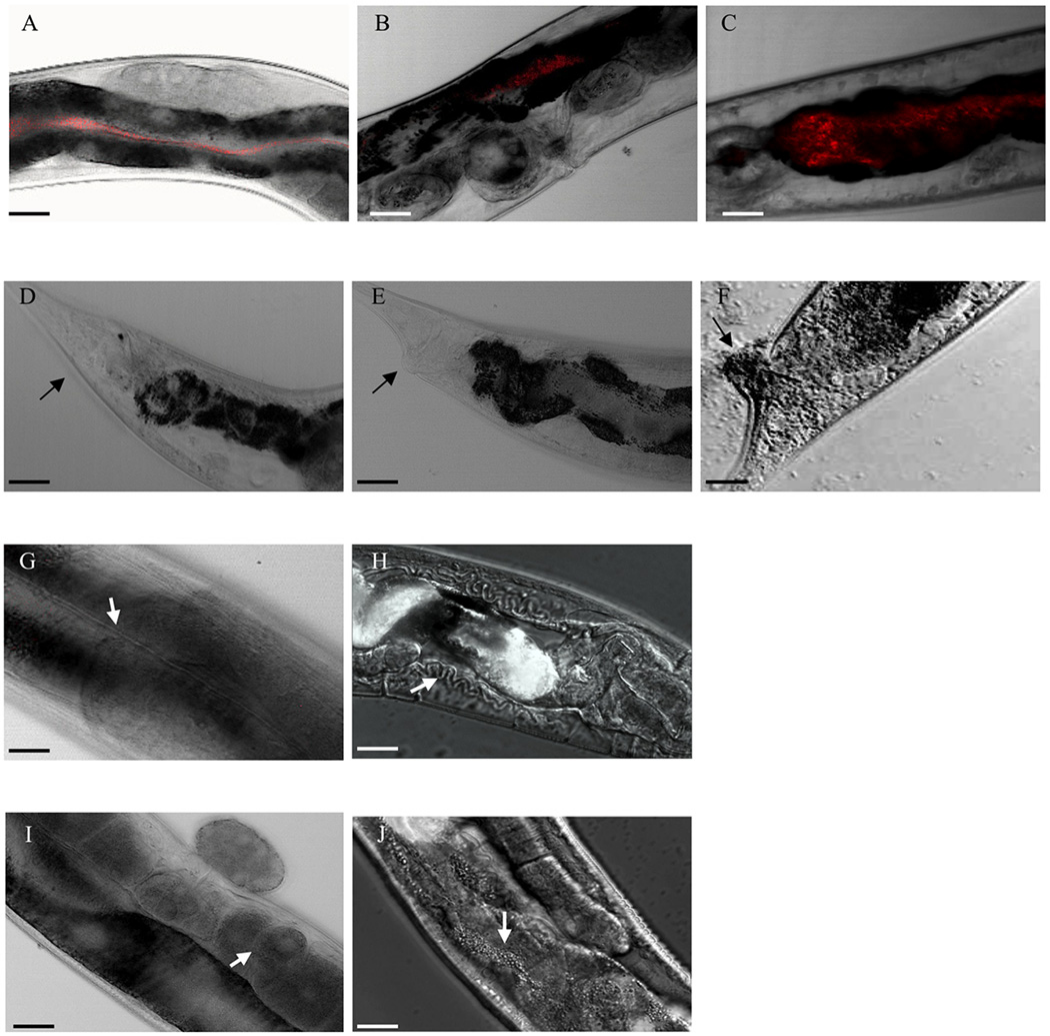
Microscopic analysis of *Caenorhabditis elegans* propagated on *Pseudomonas chlororaphis* PA23. *C*. *elegans* was grown on PA23 and its derivatives harboring the mCherry red fluorescent protein (RFP) gene on pMCh-23. Nematodes were found to exhibit assimilation of bacteria but no colonization (A), mild colonization (B), or extensive colonization (C) depending on the bacterial strain tested. *C*. *elegans* was examined for the following pathological indicators: swollen tail; enlarged excretory canals; disintegrated gonads. Panels D, E and F reveal normal, mildly swollen and extensively swollen tails, respectively (black arrows). Panels G and H depict healthy and enlarged excretory canals, respectively (white arrows). Panels I and J show healthy and disintegrated gonads, respectively (white arrows). Images shown in Panels A—E, G and I were taken using bright-field microscopy; those in Panels F, H and J were taken using differential interference contrast microscopy. Scale bar represents 25 μm.

Next, we employed scanning confocal laser microscopy to reveal the extent of colonization and pathological effects in the nematodes (Fig 4). Pathological indicators included the presence of a swollen tail (Fig 4E and 4F), enlarged excretory canals (Fig 4H and 4I), and disintegrated gonads (Fig 4J). After 72 hours, the wild type and the Δ*phz*, Δ*rpoS* and Δ*psrA* mutants all showed extensive colonization of the nematode gastrointestinal tract (Table 1). The highest incidence of swollen tails (50%), enlarged excretory canals (40%), and disintegrated gonads (30%) was found in *C*. *elegans* colonized with the *phz* mutant. These traits were observed to a lesser degree in nematodes colonized by the Δ*rpoS* and Δ*psrA* strains (Table 1). Reduced colonization by the *prn* knock out strains was accompanied by the absence of swollen tails and enlarged canals; however, disintegrated gonads were observed in 20% (Δ*prn*) and 17% (Δ*prn/phz*) of the nematodes (Table 1). Collectively, these findings indicate that PRN production facilitates PA23 colonization and leads to increased pathology in *C*. *elegans*. The Δ*hcn* strain, which also demonstrated limited colonization, induced swollen tails (10%) and enlarged excretory canals (20%) but did not impact the gonads. At 144h, there were no surviving nematodes on lawns of the wild type or the *phz*, *rpoS* and *psrA* mutants (data not shown), further establishing the impact of elevated PRN production on nematode lethality. At this point, colonization by the *prn* and *prn/phz* mutants became more extensive with some of the nematodes exhibiting swollen tails and disintegrated gonads (Table 2). The *hcn* mutant showed no changes in colonization or pathology compared to what was observed at 48 hours, while the QS-deficient strains began to colonize but they exhibited no adverse effects on the nematode tissues (Table 2). It was only after 88 hours that the *gacS* mutant showed limited colonization with no accompanying pathological changes (data not shown).

**Table 2:** Pathological symptoms of *Caenorhabditis elegans* (N2) on lawns of PA23 and derivative strains at 88h on nematode growth medium.

Strains	Time of 100%	Colonization	Swollen tail	Enlarged	Disintegrated
	Mortality (h)		(%)^1^	excretory	gonads (%)^1^
				canals (%)^1^	
PA23^a^	72	-	-	-	-
*Δphz* ^a^	64	-	-	-	-
*ΔrpoS* ^a^	72	-	-	-	-
*ΔpsrA* ^a^	72	-	-	-	-
*Δprn*	96	extensive^b^	36.8 (2.9)	0	30.5 (3.7)
Δ*prn*/*phz*	96	extensive^b^	29.9 (4.2)	0	17.8 (2.3)
Δ*hcn*	120	mild^c^	11.0 (4.0)	20.0 (3.3)	0
AI-deficient	184	mild^c^	0	0	0
Δ*phzR*	184	mild^c^	0	0	0
Δ*gacS*	208	none	0	0	0
*E*. *coli* OP50	200	none	0	0	0

^1^Twenty nematodes were examined for each strain; mean (SD) obtained from three trials.

^a^ viable nematodes were not present at 88h; consequently, colonization and pathology was not determined.

^b^extensive—colonization throughout the entire gut.

^c^mild—colonization in only specific areas of gut, either upper or lower intestine.

### Binary choice assays

**Fig 5:**
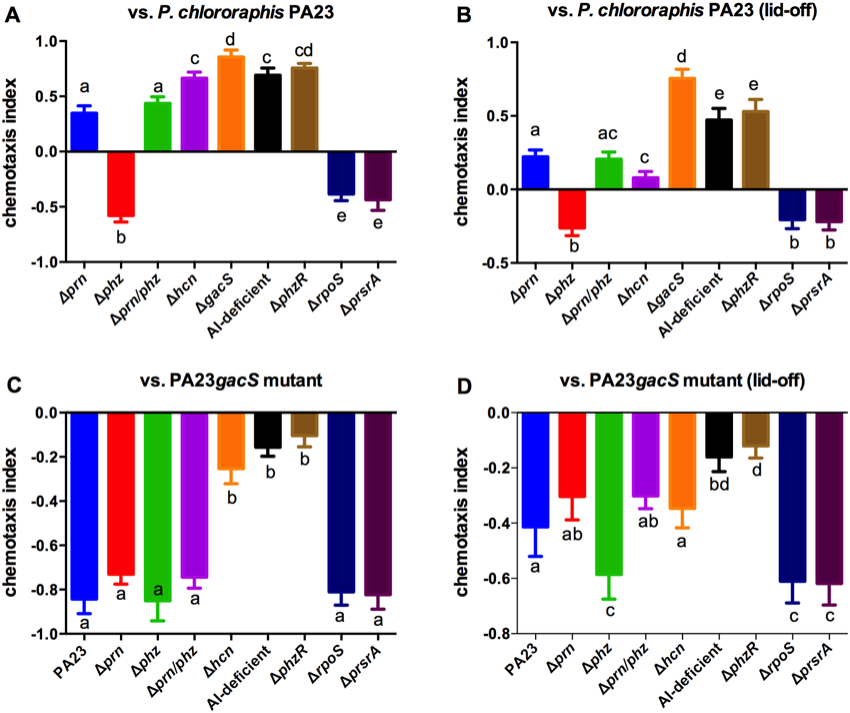
Pyrrolnitrin and hydrogen cyanide are repellents for *Caenorhabditis elegans*. Nematode preference was assessed against the wild-type strain PA23 (A) and the non-toxic strain Δ*gacS* (B). Chemotaxis was monitored by inoculating an NGM plate with the two bacterial strains to be tested. After 24 h, nematodes (50–100) nematodes were spotted on the centre of each plate. The number of nematodes at each bacterial colony was counted after 24h. The chemotaxis index was calculated as (number on spot 2—number on spot 1)/total number of nematodes at both spots. Assays were performed with the petri plate lids on (A,C) and off (B,D). Error bars indicate ± standard error; letters represent statistical groupings of means compared to the same reference strain (95% confidence, Bonferroni test).

Bacterial exoproducts can act as either attractants or repellants which in turn impacts nematode grazing. To understand how secondary metabolites produced by PA23 affect chemotactic traits of *C*. *elegans*, binary choice assays were performed. Nematodes were able to choose between colonies of the control strain, either the PA23 wild type (Fig 5A and 5B) or the *gacS* mutant (Fig 5C and 5D), and the test strain. In choice assays employing PA23 as the control, PRN was found to have a powerful repellent effect. As outlined in Fig 5A, nematodes preferred the *prn* mutants (Δ*prn* and Δ*prn/phz*) over with wild type; while the PRN overproducing strains (Δ*phz*, Δ*rpoS* and Δ*psrA*), all exhibited repulsive properties. HCN was also found to repel the nematodes, with the *hcn* mutant being preferred over the wild type. The QS-deficient strains and *gacS* mutant, which all produce little to no PRN, HCN and PHZ, were highly attractive to the animals (Fig 5A). As HCN is a volatile compound, we were interested to learn whether removing the lids from the petri plates would impact chemotaxis. While the overall pattern of chemotaxis remained the same, a few differences emerged. First, the nematodes were less attracted to the Δ*hcn* strain, which is not surprising since the bulk of the volatile HCN produced by PA23 would presumably have escaped. Second, for most of the test strains, the scale of the chemotaxis index was decreased indicating that the attractive/repulsive forces were minimized through volatile release. When the same studies were performed using the *gacS* mutant as the control, in all instances this bacterium was preferred over the test strain (Fig 5C and 5D). No differences were observed between the Δ*prn* mutant, the wild type and the PRN overproducing strains; however, the AI-deficient, the Δ*phzR* and the Δ*hcn* mutant were found to have a less repulsive effect (Fig 5C). Removal of the petri plate lids lead to some interesting changes in the pattern of chemotaxis (Fig 5D). Now the PRN overproducers had the greatest repulsive effect, while the *hcn* mutant exhibited similar repulsion to the wild type. Thus it appears that both PRN and HCN act as repellents for *C*. *elegans* with the impact of the latter being mitigated in situations where the gases are not contained.

### Growth in the presence of *Caenorhabditis elegans* affects PA23 gene expression

**Fig 6:**
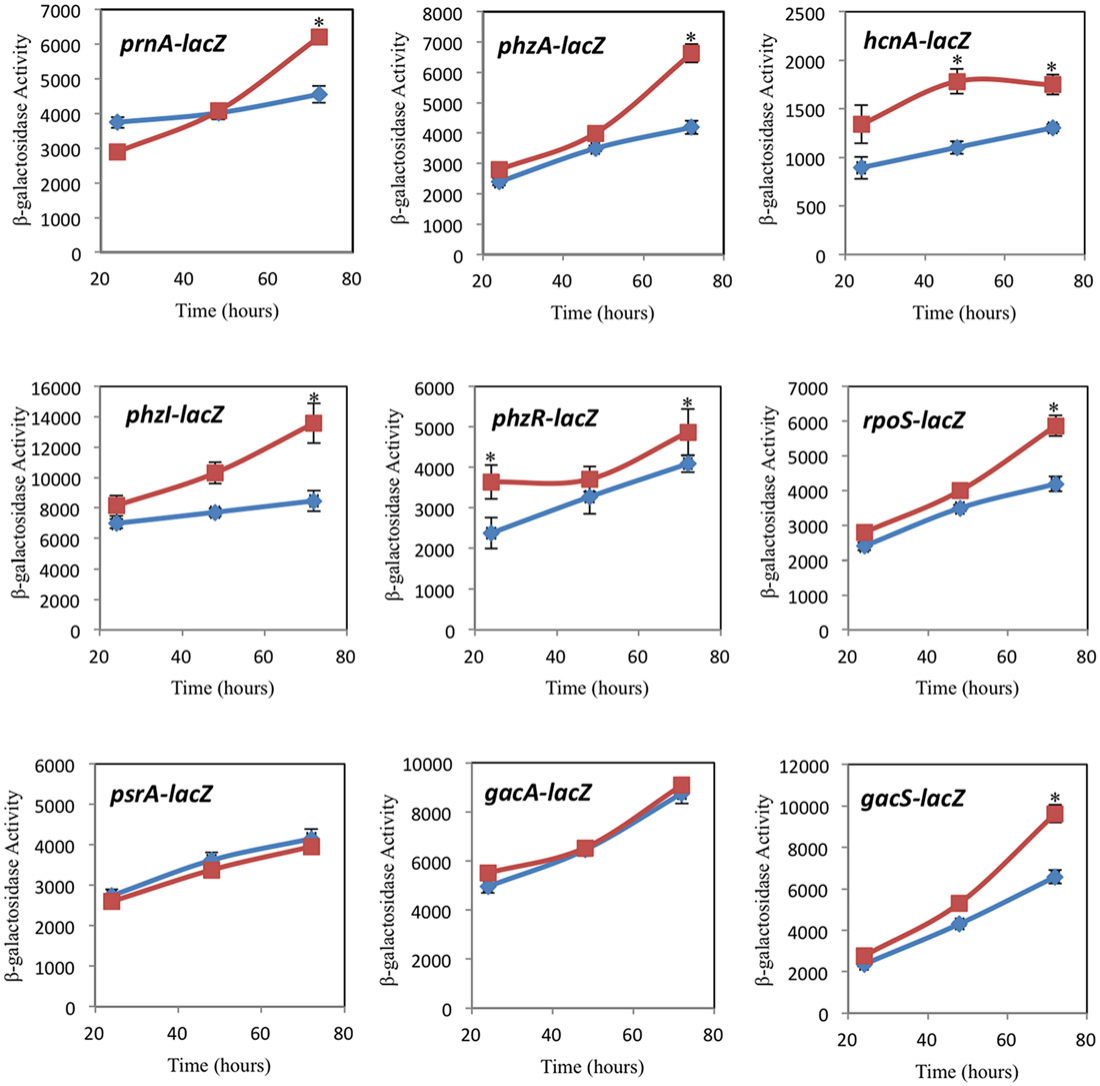
Co-culturing with *Caenorhabditis elegans* impacts *Pseudomonas chlororaphis* PA23 gene expression. PA23 cultures harboring *prnA-lacZ*, *phzA-lacZ*, *hcnA-lacZ*, *phzI-lacZ*, *phzR-lacZ*, *rpoS-lacZ*, *psrA-lacZ*, *gacA-lacZ*, and *gacS-lacZ* fusions were grown in the presence (red squares) and absence (blue diamonds) of the nematodes. Cells were assayed for β-galactosidase activity at 24, 48 and 72h. Each value represents the mean from three biological replicates ± standard error. Data points marked with an asterisk (*) are statistically significant. Experiments were performed three times; one representative data set is shown.

To investigate whether chemical cues from *C*. *elegans* are perceived by PA23, we monitored the influence of *C*. *elegans* co-culturing on bacterial gene expression. Both biosynthetic (*prnA*, *phzA*, *hcnA*) and regulatory genes (*phzI*, *phzR*, *rpoS*, *psrA*, *gacS*, *gacA*) were analyzed. As outlined in Fig 6, the presence of *C*. *elegans* lead to increased *phzA* gene expression and the magnitude of difference increased over time. For *prnA*, a different pattern of expression was observed. Initially at 24 h, *prnA* transcription was lower in *C*. *elegans* co-cultured bacteria but by 72 h, gene activity was significantly higher than in cultures containing bacteria alone (Fig 6). For *hcnA*, expression was consistently elevated in the presence of the nematodes. We next examined key regulatory genes involved in PA23 biocontrol, including the QS genes *phzI* and *phzR*. We observed increasing levels of *phzI* activity in the presence of *C*. *elegans*, with statistically significant differences observed at 48 and 72 h; whereas *phzR* showed elevated gene expression at 24 and 72h (Fig 6). Increased expression of *rpoS* was found at 72 h; whereas *psrA*, which encodes an activator of *rpoS*, remained unchanged in the presence of *C*. *elegans* (Fig 6). A similar pattern was observed for *gacS* and *gacA* with the former showing elevated expression at 48 and 72 hours in the presence of the nematodes while *gacA* exhibited no change in gene activity (Fig 6).

### Analysis of the impact of nematode co-culture on PA23 phenotypic traits

Co-culturing with *C*. *elegans* lead to elevated *phzA* and *prnA* gene expression in PA23, suggesting that antibiotic production might be upregulated in a similar fashion. As outlined in Table 3, growth in the presence of the nematodes lead to increased levels of both PHZ and PRN; however, only the former showed a significant rise. *phzI* encodes an AHL synthase responsible for synthesizing AHL molecules. Employing a bioreporter assay, we discovered that PA23 cultures grown with *C*. *elegans* produce elevated levels of the QS signalling molecules (Table 3). Thus, our end-product analysis mirrored what was observed for *phzA-*, *prnA-* and *phzI-lacZ* expression. Other phenotypic traits including fungal inhibition, protease activity and swimming motility were unaffected by the nematodes (Table 3).

**Table 3:** Phenotypic characterization of *Pseudomonas chlororaphis* PA23 grown in the presence and absence of *Caenorhabditis elegans*.

Organism	PRN^1^	PHZ^1^	AHL^1^	Antifungal^2^	Protease^2^	Motility^2^
	(μg/ml)	(μg/ml)	(Miller units)	(mm)	(mm)	(mm)
PA23	3.9 (0.5)	27.7 (0.45)	1296.7 (63.4)	5.76 (0.18)	5.4 (0.5)	71.4 (2.3)
PA23 + *C*.	5.1 (0.5)	33.9 (1.16)^a^	1932.9 (81.6)^a^	5.9 (0.16)^a^	5.5 (0.7)	75.6 (1.5)
*elegans*						

^1^Mean (SD) from three replicates.

^2^Mean (SD) of zones of activity (mm) obtained from six replicates.

^a^Significantly different from the wild type (P<0.05).

## Discussion

The ability to avoid predation either through repulsive forces or reducing predator abundance is expected to improve the success of a biocontrol agent by increasing environmental persistence. The focus of the current study was to investigate the interaction between biocontrol strain PA23 and the bacterivorous nematode *C*. *elegans*; in particular, we were interested to learn whether PA23 demonstrates nematicidal and or repellent activities.

On nutrient-rich BHI media, which supports rapid growth and production of high levels of secondary metabolites, strains deficient in HCN expression (*hcn* and *gacS* mutants) were unable to induce rapid death of *C*. *elegans* (Fig 1). The highest degree of nematicidal activity was observed for the PRN overproducing strains (Fig 1). Collectively, these findings indicate that HCN is the primary compound involved in *C*. *elegans* intoxication (Fig 1); however, at elevated levels, PRN also exhibits toxic effects. In slow-killing assays, which depend upon infection of the *C*. *elegans* intestine, a deficiency in either HCN or PRN production lead to decreased killing. While HCN is well established as inducing lethal paralysis in *C*. *elegans* [8], to the best of our knowledge this is the first study to report nematicidal activity associated with PRN. To further confirm its toxic effects, L4-stage adults were incubated in the presence of increasing concentrations of purified PRN. We observed that PRN exposure lead to reduced viability in a dose-dependent manner (Fig 3A). In a study by Meyer and colleagues [29], purified 2,4-diacylphloroglucinol (DAPG) exhibited toxic effects towards adults of the plant-parasitic nematode *Xipinema americanum* but did not affect *C*. *elegans* J1 or adult-stage nematodes. Interestingly, 1- and 3-hour incubation with DAPG actually stimulated *C*. *elegans* egg hatch [29]. In the current study, we observed a reduction in the frequency of egg hatching upon *C*. *elegans* propagation on the PRN-overproducing strains (Table 1) as well as when eggs were incubated in a PRN solution (Fig 3B). At lower concentrations, egg hatching was merely delayed; however at higher, physiologically relevant concentrations (1, 5 and 10 μg/ml), hatching decreased to less than 50% of wild type after 24h exposure (Fig 3B). Collectively, these findings indicate that PRN affects many aspects of *C*. *elegans* physiology, acting as a nematicide and repellent for adult nematodes and reducing egg hatching.

Although PA23 produces two PHZ compounds, namely PCA and 2-OH-PHZ, they do not appear to be important for nematicidal activity. Under fast- and slow-killing conditions, there was no difference in lethality between the *prn* mutant and the *prn/phz* double mutant (Figs 1 and 2). If PHZ was contributing in some way to overall lethality, we would expect a reduction in mortality associated with the *phz* mutants, both of which are devoid of PHZ production [17]. The fact that the single *phz* mutant exhibits the highest rate of killing in both the fast- and slow-killing assays supports the notion that PHZs are not involved in these two processes. Similar findings were reported for *P*. *aeruginosa* strain PAO1, wherein PHZs did not impact fast-killing by bacteria propagated on BHI media [3]. For *P*. *aeruginosa* PA14 grown on PGS agar, PCA was reportedly the primary compound underlying intoxication, with increased toxicity observed at lower pH ranges [7]. Differences in media (PGS versus BHI) could account for the discrepancy in findings between these studies. It is also important to note that strain PA14 produces nearly twice as much PCA [52.7 μg/ml; 7] as PA23 [28.5 μg/ml; 17] which may have contributed to the observed differences as well. The notion that antibiotic concentration significantly impacts *C*. *elegans* viability is supported by our fast-killing assays, wherein the PRN overproducing strains exhibited elevated toxicity while the *prn*-null mutants were unaffected compared to PA23 (Fig 1).

The ability to avoid grazing-predator interactions all together would presumably benefit biocontrol bacteria to a greater extent than nematicidal activity. When we analyzed PA23 repellence of *C*. *elegans*, both HCN and PRN were able to act as powerful repellents (Fig 5). In a study by Burlinson et al., [6], screening of a *Pseudomonas fluorescens* NZ17 transposon library revealed several genetic loci associated with *C*. *elegans* repellence. Among these were *gacS* and a newly-identified cluster of genes named EDB, for edible. While the EDB cluster was found to be under GacS control, the mechanism underlying EDB-mediated repellence is currently unknown [6]. Analysis of the PA23 genome failed to reveal EDB homologues (data not shown). Much like what was observed in the current study, the NZ17 *gacS* mutant exhibited the lowest repellence, while mutants deficient in the production of a single exoproduct (HCN, TOL, exoenzymes) retained some repellent activity [6].

Chemical signalling plays an important role in the interaction of an organism with its environment. Because bacterial exoproducts modulate the PA23-*C*. *elegans* interaction through their nematicidal and repellent effects, we were interested to learn whether the presence of *C*. *elegans* would elicit changes in PA23 gene activity. These changes could be mediated by either direct contact with the nematode or through perception of soluble chemical cues. Our analysis of regulatory genes revealed altered expression in some but not all cases. In terms of biosynthetic genes, co-culturing of the two organisms lead to increased *prnA*, *phzA* and *hcnA* gene expression at 72 h compared to growth in the absence of *C*. *elegans* (Fig 6). Exoproduct analysis showed that PHZ and AHL were significantly upregulated in the presence of *C*. *elegans*. PRN is the primary antibiotic responsible for PA23-mediated suppression of *S*. *sclerotiorum* [17]; therefore, the unaltered change in antifungal activity upon co-culture with *C*. *elegans* was not surprising considering that PRN production was not significantly elevated. Jousset and coworkers [11] reported similar findings wherein *P*. *fluorescens* CHA0 grown in the presence of cell-free supernatants of the amoeba *Acanthamoeba castellani* exhibited elevated *phlA* (DAPG), *prnA* and *hcnA* gene expression and increased DAPG, PRN and HCN production. However, in direct contrast to our findings, these researchers observed that co-culturing with *A*. *castellani* decreased gene expression [11]. It was concluded that in response to soluble predator cues, CHA0 upregulates defense mechanisms; however, direct contact with bacteria enables the amoeba to repress bacterial toxicity [11]. In a second study, the cyclic lipopeptides massetolide and viscosin produced by *P*. *fluorescens* strains SS101 and SBW25, respectively, were found to protect bacteria from *Naegleria americana* protozoan grazing [10]. When bacteria were either in direct contact with or in close proximity to *N*. *Americana*, increased *massABC* (massetolide) and *viscABC* (viscosinamide) expression was observed. Collectively these findings indicate that bacteria and bacterial-feeding eukaryotes are able to sense one another through soluble chemical cues and/or direct contact and this mutual perception modulates the predator-prey interaction. The idea of inter-kingdom signalling between bacteria and higher eukaryotes is not new. Much of the research to date has focused on quorum-sensing signals as the basis for this communication [30]. Recent findings suggest that the antibiotics serve multiple dose-dependent functions. At higher concentrations, they can inhibit or kill competing microbes, while at lower levels they act as intercellular signals capable of modulating bacterial gene expression [31,32]. Our findings and those of others [10,11] suggest that antibiotics may represent another “language” of communication between bacteria and eukaryotic organisms.

In summary, HCN and PRN are key compounds that affect the interaction of PA23 and *C*. *elegans*. HCN is well established as being toxic to *C*. *elegans*; however, our finding that PRN is a nematicidal agent is novel. Interestingly, PRN is the most important antibiotic for PA23-mediated biocontrol of *S*. *sclerotiorum*. As environmental persistence is an essential feature of a successful biocontrol agent, PRN appears to play a role that extends beyond PA23-mediated pathogen suppression. Because synthesis of antifungal compounds is energetically costly, it makes sense for bacteria to limit production of these compounds to situations where they impart a fitness advantage. The presence of *C*. *elegans* leads to altered PA23 gene expression, indicating the bacteria are able to perceive soluble chemical cues and/or direct nematode contact, and this in turn modulates PA23 physiology. Studies are on-going to further define the mechanisms involved in sensing chemical signals in both *C*. *elegans* and PA23. Future work will seek to determine whether PA23 exhibits broad-spectrum nematicidal and repellent activities against a range of nematodes, including plant-pathogenic organisms, under both lab and field conditions.

## Supporting Information

S1 Fig Model for the regulatory network overseeing production of *Pseudomonas chlororaphis* PA23 antifungal factors. Evidence for the proposed pathway comes from previous studies [16,18,19,20]. In response to an unknown signal, the sensor kinase GacS undergoes autophosphorylation and phosphotransfer to the response regulator GacA. Activated GacA induces expression of the non-coding RNA RsmZ, the post transcriptional repressors RsmA and RsmE, and the sigma factor RpoS. RpoS is under positive control of PsrA and the SR. RpoS activates expression of *phzI*, but represses *phzR* and the pyrrolnitrin biosynthetic genes. The Phz QS system positively regulates *rpoS* as well as the phenazine, pyrrolnitrin and HCN biosynthetic loci. Symbols: ↓, positive effect; ⊥, negative effect; solid lines, direct effect; broken lines, indirect effect.
(TIFF)

S2 Fig Hydrogen cyanide is under quorum sensing control in *Pseudomonas chlororaphis* PA23. Hydrogen cyanide production by the PA23 wild type (panel A), *phzR* mutant (panel B) and AI-deficient strain (panel C) was assessed using cyantesmo paper, which turns blue in the presence of HCN. Note the reduced HCN production by the two quorum-sensing deficient strains compared to the wild type.
(TIFF)

S1 Table Bacterial strains, plasmids, and primers used in the study. (DOCX)
