# Supplementary figures and images for: Pyrrolnitrin and Hydrogen Cyanide Production by Pseudomonas chlororaphis Strain PA23 Exhibits Nematicidal and Repellent Activity against Caenorhabditis elegans
Source: PLoS One. 2015 Apr 22;10(4):e0123184. doi: 10.1371/journal.pone.0123184 (PMC4406715; doi:10.1371/journal.pone.0123184)

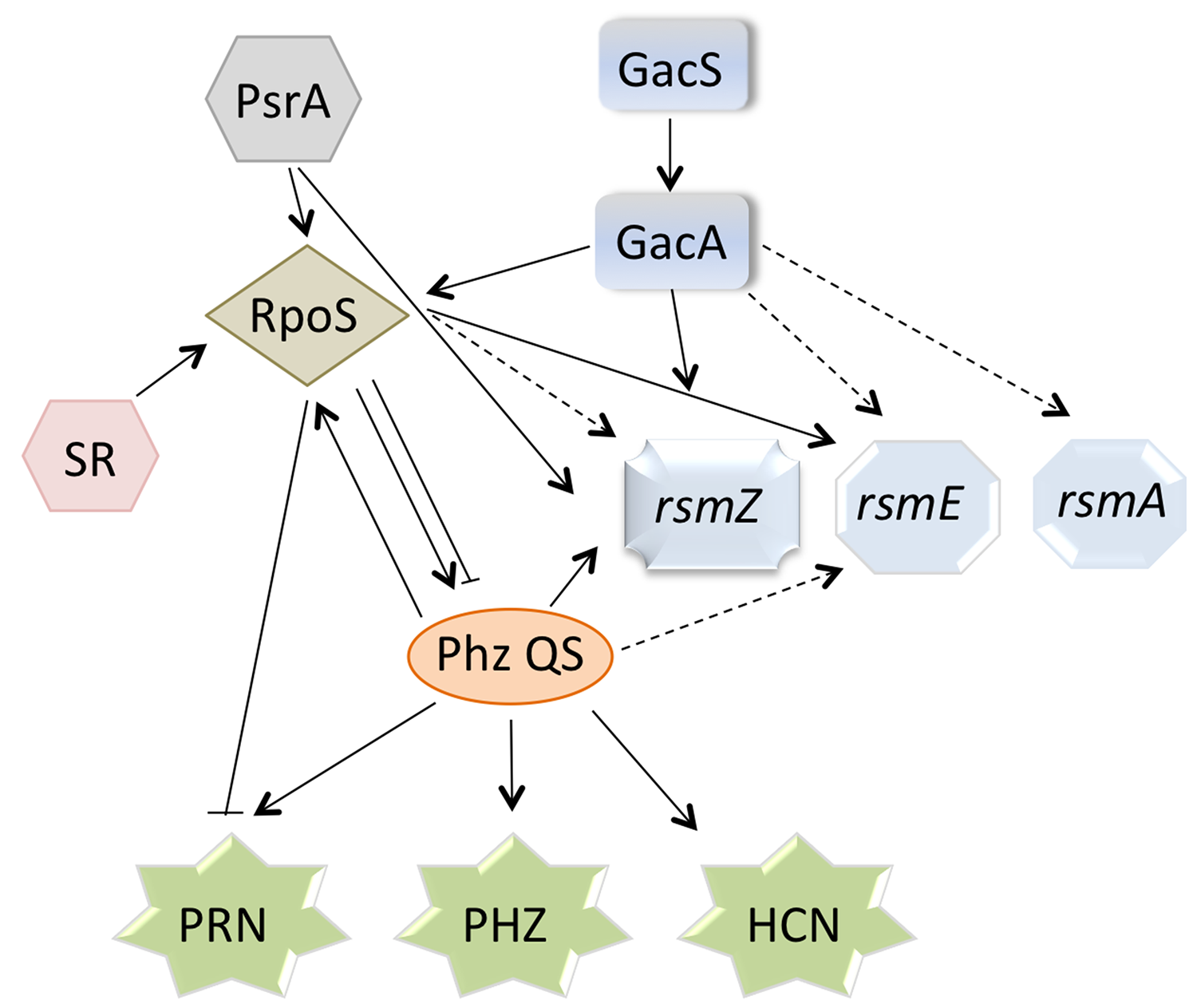

Supplement: S1 Fig — Evidence for the proposed pathway comes from previous studies [16,18,19,20]. In response to an unknown signal, the sensor kinase GacS undergoes autophosphorylation and phosphotransfer to the response regulator GacA. Activated GacA induces expression of the non-coding RNA RsmZ, the post transcriptional repressors RsmA and RsmE, and the sigma factor RpoS. RpoS is under positive control of PsrA and the SR. RpoS activates expression of phzI, but represses phzR and the pyrrolnitrin biosynthetic genes. The Phz QS system positively regulates rpoS as well as the phenazine, pyrrolnitrin and HCN biosynthetic loci. Symbols: ↓, positive effect; ⊥, negative effect; solid lines, direct effect; broken lines, indirect effect. (TIFF) [file pone.0123184.s001.tiff]

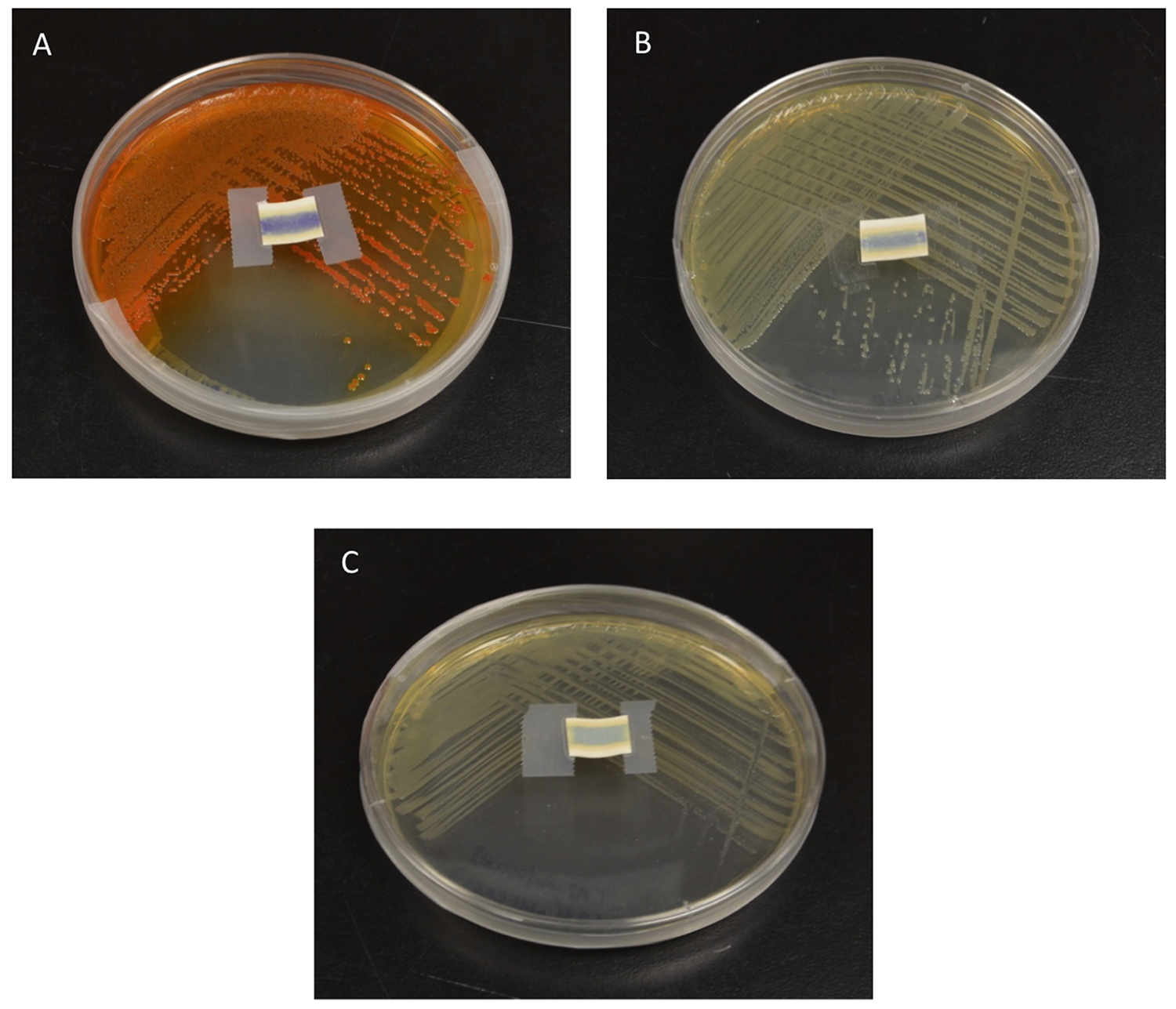

Supplement: S2 Fig — Hydrogen cyanide production by the PA23 wild type (panel A), phzR mutant (panel B) and AI-deficient strain (panel C) was assessed using cyantesmo paper, which turns blue in the presence of HCN. Note the reduced HCN production by the two quorum-sensing deficient strains compared to the wild type. (TIFF) [file pone.0123184.s002.tiff]
