# Supplementary material for: Pyrrolnitrin and Hydrogen Cyanide Production by Pseudomonas chlororaphis Strain PA23 Exhibits Nematicidal and Repellent Activity against Caenorhabditis elegans
Source: PLoS One. 2015 Apr 22;10(4):e0123184. doi: 10.1371/journal.pone.0123184 (PMC4406715; doi:10.1371/journal.pone.0123184)
Supplement: S1 Table — (DOCX) [file pone.0123184.s003.docx]

**Supplemental Table 1.** Bacterial strains, plasmids, and primers used in the study.

| **Strain, plasmid, or Primer** | **Relevant genotype, phenotype or sequence** | **Reference or source** |
| --- | --- | --- |
| **Strains**  *P. chlororaphis*  PA23 | PRN^+^PHZ^+^Rif^R^; wild-type (soybean root tip isolate) | [14] |
| PA23-8 | PRN^-^Rif^R^ *prnBC* deletion mutant | [17] |
| PA23-63 | PHZ^-^Rif^R^ *phzE*::Tn5-OT182 genomic fusion | [17] |
| PA23-63-1 | PRN^-^PHZ^-^Rif^R^ *phzE*::Tn5-OT182 genomic fusion; *prnBC* deletion mutant | [17] |
| PA23*hcn* | PA23 with the pKNOCK-Tc vector inserted into the *hcn* gene | This study |
| PA23-6863 | PA23 carrying pME6863; AHL deficient | [19] |
| PA23*phzR* | PA23 with Gm^R^ marker inserted into *phzR* gene | [19] |
| PA23*rpoS* | PA23 with pKNOCK-Tc vector inserted into *rpoS* gene | [19] |
| PA23*psrA* | PA23 with pKNOCK-Tc vector inserted into *psrA* gene | [18] |
| PA23*gacA* | Gm^R^ marker inserted into the *gacA* gene | [18] |
| PA23-314 | Rif^R^ *gacS*::Tn-OT182 genomic fusion | [16] |
| PA23-*rfp* | PA23 containing mCherry expressed from pMCh-23 | This study |
| PA23-8-*rfp* | PA23-8 containing mCherry expressed from pMCh-23 | This study |
| PA23-63-*rfp* | PA23-63 containing mCherry expressed from pMCh-23 | This study |
| PA23-63-1-*rfp* | PA23-63-1 containing mCherry expressed from pMCh-23 | This study |
| PA23*hcn*-*rfp* | PA23*hcn* containing mCherry expressed from pMCh-23 | This study |
| PA23-6863-*rfp* | PA23-6863 containing mCherry expressed from pMCh-23 | This study |
| PA23*phzR*-*rfp* | PA23*phzR* containing mCherry expressed from pMCh-23 | This study |
| PA23*rpoS*-*rfp* | PA23*rpoS* containing mCherry expressed from pMCh-23 | This study |
| PA23*psrA*-*rfp* | PA23*psrA* containing mCherry expressed from pMCh-23 | This study |
| PA23*gacS*-*rfp* | PA23*gacS* containing mCherry expressed from pMCh-23 | This study |
| *P. aeruginosa*  QSC105 | Strain carrying pEAL01 (*lasB-lacZ* transcriptional fusion), Carb^R^ | [28] |
| *E. coli*  DH5α | *supE44* Δ*U169* (*Φ*80*lacZ*ΔM15) *hsdR17 recA1 endA1 gyrA96 thi-1 relA1* | Gibco |
| DH5α λpir | λpir lysogen of DH5α | ^a^ |
| OP50 | Laboratory strain for maintenance of *C. elegans*. Uracil auxotroph. | ^b^ |
| *Chromobacterium violaceum* CVO26 | Autoinducer synthase (*cviI*) mutant from *C. violaceum* ATCC 31532 autoinducer biosensor | ^c^ |
| **Plasmids**  pME6863 | pME6000 carrying the *aiiA* gene from *Bacillus* sp.A24 under the constitutive P_lac_ promoter | ^d^ |
| pCR2.1 | TA cloning vector, Amp^R^ | Invitrogen |
| pKNOCK-Tc | Suicide vector for insertional mutagenesis; R6K ori Rp4 oriT Tc^R^ | [25] |
| pRK600 | Contains *tra* genes for mobilization, Chl^R^ | ^e^ |
| pCR*hcnABC* | 1.9-kb *hcnABC* fragment in pCR2.1 | This study |
| pKNOCK-*hcnABC* | 1.9-kb fragment from *hcnABC* in pKNOCK-Tc | This study |
| pUCP23 | Broad-host-range vector, Amp^R^Gm^R^ | ^f^ |
| pMCh-23 | pUCP23 carrying the mCherry red fluorescent protein gene | [26] |
| pLP170 | *lacZ* transcriptional fusion vector | ^g^ |
| pPRNA-*lacZ* | *prnA* promoter in pLP170 | [17] |
| pPHZA-*lacZ* | *phzA* promoter in pLP170 | [17] |
| pPHZI-*lacZ* | 674-bp fragment containing the *phzI* promoter in pLP170 | [19] |
| pPHZR-lacZ | 1.1-kb fragment containing the *phzR* promoter in pLP170 | [19] |
| pRPOS-*lacZ* | *rpoS* promoter in pLP170 | [16] |
| pCR-*psrA* | 948-bp *psrA* gene in pCR2.1 | This study |
| pPSRA-*lacZ* | *psrA* promoter in pLP170 | This study |
| pCR*gacS*up | *gacS* promoter in pCR2.1 | This study |
| pGACS-*lacZ* | *gacS* promoter in pLP170 | This study |
| pCR*gacA*up | *gacA* promoter in pCR2.1 | This study |
| pGACA-*lacZ* | *gacA* promoter in pLP170 | This study |
| pME3219 | pME6010 containing an *hcnA-lacZ* translational fusion | ^h^ |
| **Primers**  hcnA-FOR | 5′- atgcggacatgaccatcagc-3′ | This study |
| hcnC-REV | 5′-aatccaccaccaccgccgaacc-3′ | This study |
| nGacAtrans-FRW | 5′-gaagatcttcttaagacaggaaagggaacgc-3′ | This study |
| nGacAtrans-REV | 5′-ccaagcttgggacttgcaggccatcgatgt-3′ | This study |
| newGacStrans-FRW | 5′-ggggtaccccgggattcattagcttctgcaa-3′ | This study |
| newGacSRtrans-REV | 5′-cgggatccccgattcccaaatggttgagcaca-3′ | This study |
| psrAFOR | 5′-ctcttggcaatcctcct-3′ | This study |
| psrAREV | 5′-ttagcggatgtaagctgc-3′ | This study |
| psrA*Bam*HI-FRW | 5′-ccggatccggtgacgccggtttca-3′ | This study |
| M13-REV | 5′-caggaaacagctatgac-3′ | Invitrogen |

Rif, rifampicin; Tc, tetracycline; Gm, gentamycin; Carb, carbenicillin; Chl, chloramphenicol; Amp, ampicillin.

^a^House BL, Mortimer MW, Kahn ML (2004) New recombination methods for *Sinorhizobium meliloti* genetics. Appl Environ Microbiol 70: 2806-2815.

^b^Latifi A, Foglino M, Tanaka M, Williams P, Ladzdunski A (1996) A hierarchial quorum-sensing cascade in *Pseudomonas aeruginosa* links the transcriptional activators LasR and RhlR (VsmR) to expression of the stationary phase sigma factor RpoS. Mol Microbiol 21: 1137-1146.

^c^Reimmann C, Ginet N, Michel L, Keel C, Michaux P, Krishnapillai V, et al. (2002) Genetically programmed autoinducer destruction reduces virulence gene expression and swarming motility in *Pseudomonas aeruginosa* PAO1. Microbiology 148: 923-932.

^d^Finan T M, Kunkel B, Vos G F D, Signer ER (1986) Second symbiotic megaplasmid in *Rhizobium meliloti* carrying exopolysaccharide and thiamine synthesis genes. J Bacteriol 167: 66-72.

^e^West SE, Schweizer HP, Dall C, Sample, AK, Runyen-Janecky LJ (1994) Construction of improved *Escherichia-Pseudomonas* shutle vectors derived from pUC18/19 and sequence of the region required for their application in *Pseudomonas aeruginosa.* Gene 148: 81-86.

^f^Preston MJ, Seed PC, Toder DS, Iglewski BH, Ohman DE, Gustin JK, et al. (1997) Contribution of proteases and LasR to the virulence of *Pseudomonas aeruginosa* during corneal infections. Infect Immun 65: 3086-3090.

**^g^Laville J, Voisard C, Keel C, Maurhofer M, Défago G, Haas.** **D (**1992) Global control in Pseudomonas fluorescens mediating antibiotic synthesis and suppression of black rot of tobacco. Proc Natl Acad Sci USA 89: 1562-1566.
